# Energy Aware Software Defined Network Model for Communication of Sensors Deployed in Precision Agriculture

**DOI:** 10.3390/s23115177

**Published:** 2023-05-29

**Authors:** Shakeel Ahmed

**Affiliations:** Department of Computer Science, College of Computer Sciences and Information Technology, King Faisal University, Al-Ahsa 31982, Saudi Arabia; shakeel@kfu.edu.sa

**Keywords:** precision agriculture, software defined network, network latency, network lifetime, sensor nodes, throughput

## Abstract

A significant technological transformation has recently occurred in the agriculture sector. Precision agriculture is one among those transformations that largely focus on the acquisition of the sensor data, identifying the insights, and summarizing the information for better decision-making that would enhance the resource usage efficiency, crop yield, and substantial quality of the yield resulting in better profitability, and sustainability of agricultural output. For continuous crop monitoring, the farmlands are connected with various sensors that must be robust in data acquisition and processing. The legibility of such sensors is an exceptionally challenging task, which needs energy-efficient models for handling the lifetime of the sensors. In the current study, the energy-aware software-defined network for precisely selecting the cluster head for communication with the base station and the neighboring low-energy sensors. The cluster head is initially chosen according to energy consumption, data transmission consumption, proximity measures, and latency measures. In the subsequent rounds, the node indexes are updated to select the optimal cluster head. The cluster fitness is assessed in each round to retain the cluster in the subsequent rounds. The network model’s performance is assessed against network lifetime, throughput, and network processing latency. The experimental findings presented here show that the model outperforms the alternatives presented in this study.

## 1. Introduction

Agriculture is defined as an activity humans undertake to ensure that a population has access to adequate, safe, and nutritious food sustainable. Many countries rely on the exports of agricultural products to generate adequate revenue to feed their citizens. In such a context, agricultural ties and crop yields are essential, and more strategies and approaches have been practiced recently to ensure productivity enhancement [1]. Real-time monitoring of the environmental condition and remote control in agriculture is fast expanding to create more profitable and efficient agricultural systems and instruments. Precision agriculture has the potential to go in this direction. These two words refer to integrating sophisticated technology with traditional agricultural techniques for fine-grid crop management. Farmers may benefit from vital environmental information from their cultivated regions provided by intelligent farming systems, enabling them to boost their productivity and revenue. These technological advancements have the potential to benefit practically every aspect of agriculture, from seeding to irrigation to crop management and harvesting systems [2].

Precision Agriculture (PA) is a movement that aims to facilitate and optimize agricultural growth for both farmers and society. It is important to note that PA is an advanced farming technology that monitors, evaluates, and records agricultural areas and crops. Advanced sensing technology has allowed on-site soil and climate monitoring to provide precise recommendations. Increased agricultural production, decreased environmental consequences, and enhanced human well-being may be achieved using Internet of Things (IoT) technology. Sensors would assist in monitoring farm inputs [3]. Farmers use optimized inputs like water and fertilizer to boost output without sacrificing quality and cost [4]. The desire for increasing agricultural efficiency while minimizing environmental impact pushes the progress of innovation towards integrated “smart” agricultural production that replace traditional farming techniques. Knowledge-based agricultural management systems with autonomous systems have been created to minimize the inputs by considering time-sensitive and demographic crop-centric conditions [5]. However, feasibility studies analyzing the costs and advantages of implementing agricultural robotic mechanisms as recently launched models are required to enable greater acceptance by sector users [6,7].

The sensor nodes would transfer the real-time data to the base station (BS), which is further processed for precise decision-making. The sensors’ lifetime and the sensor devices’ communication would make the process challenging. The data is transferred over a predefined path as the routing technique recommends. Static routing techniques are more secure than dynamic routing; nevertheless, solutions based on static routing are not suitable for broad areas like agriculture farms or in the network where scalability matters. IoT technology has been extensively fused with other industries in recent years to encourage interaction that would enhance network performance, resource usage, and load distribution [8]. Climate-related difficulties may be solved in the agriculture sector by implementing innovative IoT systems, which can boost agricultural yields and productivity. For decades, agriculture has used sensors to procure data in static settings. The offline configuration can gather static data and gives enough knowledge to make excellent judgments about future yields or crops for the following year. Still, it cannot provide data on rapid environmental changes jeopardizing agricultural products.

This article aims to use cutting-edge IoT-based sensor infrastructure to gather information from the surroundings and transfer the sensor data to the BS for timely decision-making in precision agriculture. Wireless agriculture sensors are dispersed across the agricultural field in the suggested framework to extract information relating to soil composition, such as moisture, temperature, and humidity levels. This data is safely transported to the cluster heads, which serve as the point of contact or as a manager for data exchange to the BS. Following is a list of the primary aspects of the study.

- The suggested software-defined network is efficient in establishing a wireless sensor network that is robust and computationally efficient with multiple clusters of nodes with divergent levels of residual energy.
- The sensor node’s responsibilities are delegated to the cluster heads for further processing, increasing the nodes’ survival. It ensures node availability by discharging responsibilities depending on available residual energy.
- The clusters are updated over multiple rounds concerning their fitness values, that result in clusters with an optimal number of nodes.
- Finding a cluster head (CH) that can effectively oversee data flow between sensor area nodes and the BS.
- Each round, the CH-index value is updated concerning the local and global best node index values. At the same time, the initial CH-index is determined based on the residual energy and the distance to the BS.
- Validated the proposed framework against existing studies concerning various network characteristics and summarized the findings.

The other sections of the paper are organized as follows. Section 2 discusses the Material and Methodology, which discusses different networking metrics used in evaluating the model and the assumption in the simulated network. Section 3 discusses the proposed Energy Aware Software Defined Network model. Section 4 summarizes the results obtained from the experimentation. Section 5 offers the conclusion and future scope.

### Literature Review

Placidi et al. [9] have proposed a low-cost soil moisture sensor named Sentek commercial sensor that works over a LoRa WAN-based network for economic precision agriculture. Loamy to silty soils were compared. The moisture readings were accurate. However, reliability issues were found that might be addressed by using costlier commercial sensors. The research emphasizes sensors for analyzing soil moisture content without handling other critical characteristics like pH, temperature, humidity, and sunlight. Hsu et al. [10] offer a novel service approach built on top of the IoT cloud computing platform, which may be employed to enhance the current approach of integrating the cloud-to-physical networking and the processing capabilities of the IoT. This study applies cutting-edge platform technologies to the cloud agricultural platform. It may collect vast area data and analysis through cloud integration, enabling farms with limited network bandwidth data resources to provide agricultural monitoring automation and pest control picture analysis.

A robust network infrastructure for monitoring and regulating agricultural fields in remote areas was introduced in research by Ahmed et al. [11]. They introduced an IoT-based control system for farming and agriculture development. All topological components and enhancements are thoroughly reviewed and studied. The IoT routing and MAC solution accomplished energy efficiency, low latency, and substantial throughput. Integrating a Wi-Fi long-distance (WiLD) network over a fog-computing strategy makes the system’s performance possible. Another study for alerting the farmers on mildew issues was proposed by Sergio et al. [12], using the IoT paradigm. They presented the SEnviro (Sense our Environment platform) system to monitor grape crops. To reduce communication between endpoints, they employed the edge computing concept. The authors in the study on an irrigation system based on IoT that recognizes plants automatically, Kwok et al. [13], have used deep learning to recognize the kind and category of plants for an automated plant watering system. The plant’s water need is computed by identifying a current set of plant photos and data set obtained from the farm. When the identification procedure is accomplished, it uses the database to obtain irrigation information. Modeling training procedures takes time since many photos must be saved.

In the study by Ratnaparkhi et al. [14], sensors are investigated as the most potent instrument for IoT deployment. Based on their uses, a broad range of agricultural sensors are provided. Sensor arrays, location, acoustic and airflow sensors were among the sensors studied. It was also discovered that agricultural sensors increase agricultural output. Some of the issues encountered in installing sensors in Distributed systems include their customization, continuous Wi-Fi connection, the management of errors and malfunctions, and identifying the proper sensors for diverse contexts.

Jawad et al. [15] conducted a study on agricultural applications based on wireless sensor networks. This study aims to conduct a comparative analysis of Wi-Fi, Bluetooth, LoRa, diverse cellular technologies, and Sig Fox wireless and protocol suites. LoRa and ZigBee proved effective for Precision Agriculture because of their long-range communications and low energy needs. Many methods and techniques concerning the power consumption of WSNs were grouped. The authors propose a smart agricultural IoT (SMAIoT) system to track and analyze data from various inexpensive sensors [16]. This network infrastructure is designed to gather data from soil, air, groundwater, and animals and utilize them to make appropriate judgments. The suggested framework’s distinctive feature is automating tasks, such as irrigation, fertilizers, insect identification, and pesticide spraying, with high productivity.

Long-Range Wide-Area Network (LoRaWAN) [17,18] is a technology that could internet connect multiple devices over the internet in a much-secured manner. The LoRaWAN is inspired by Low Power Wide Area Network (LPWAN) due to its capacity to communicate across large distances [19]. LoRaWANs offer low-power, low-cost, long-range, and low-data-rate communication. LoRaWAN includes end devices, gateways, network servers, and applications. Gateways and network servers link hundreds of thousands of LoRa end devices. LoRa lets low-power devices communicate long-distance with low bitrate. It is the best choice for most IoT applications, including smart towns, smart billing, intelligent transportation, automated lighting, and precision agriculture. LoRa only works in low-bitrate settings [20]. The main limitation of the LoRaWAN technology is the limited number of nodes based on the duty cycles, and not suitable for applications that rely on low latency applications. 

Vij A et al. [21] have used a pre-processed agriculture data set to add machine-intelligent approaches to the agricultural decision support model. Rather than utilizing a publicly accessible dataset, a model is designed to gradually learn plants’ watering demands. Various ML algorithms are tested in terms of accuracy for making irrigation choices. Before making accurate judgments, manual irrigations are conducted twice. Because of the model’s dynamic nature, data are processed in stages and may be used for several plants with different watering circumstances. There is a great necessity for a learning method that can be taught by itself utilizing a substantially lighter learning procedure with environmental factors, which does not require more memory in the system but require more computing. According to the study, edge computing should be included in the agricultural system for making accurate decisions with immediate calculation locally. This article will describe a smart infrastructure that uses IoT and edge computing to monitor soil moisture using sensors, data transfer among sensors, and an Analytics-as-a-Service cloud. The details of various state-of-art approaches are shown in Table 1.

The existing technologies for precision agriculture primarily focus on data acquisition and processing of the data for precise recommendations to the farmers. Some studies focus on the security aspects of the study. There are ample studies that focus on the lifetime of the network and effective communication among the nodes in the network. However, the models either focus on the survivability of the nodes or the efficient routing mechanism, not on both things simultaneously. Nevertheless, there is demand for a model to maximize the network’s lifetime and routing techniques for pushing the data across the sensor devices and the BS. The suggested software-defined network is efficient concerning the sustainability of the nodes. Additionally, the proposed model does have minimal network latency. The CH is selected based on the index values populated based on the local and global best values, resulting in an optimized way of assessing the node index values and choosing an optimal CH for the data exchange. The implementation of the proposed technology is discussed in the forthcoming sections of the study. The pivotal point of the study focuses on the survivability of the network but maintaining the throughput and latency of the network to be at the optimal level. However, the latency of the network is not a crucial parameter in PA, yet the model desired to maintain the latency to be minimal. 

## 2. Material and Methodology

IoT technologies have recently been used in various areas because of their cheap cost, ease of implementation, and cost-effective ecosystem. A vast array of sensor nodes was dispersed around the field to perceive the required data in IoT [24]. Under either a single-hop or multi-hop data transmission paradigm, data are gathered and transmitted to the BS for further processing. The current section of the paper elaborates on the details of the implementation environment, evaluation parameters used in assessing the model’s efficiency, and the proposed energy-aware software-defined network model.

The IoT environment is separated into multiple regions, and each area has one cluster leader responsible for gathering and forwarding sensory input to BS. Additionally, most sensor nodes went into sleep mode to extend the network lifespan. Various energy-aware technologies are used to formulate the network architecture for exchanging data among the nodes. Some of those technologies include Low energy adaptive cluster hierarchy (LEACH) [25], improved chain-based clustering hierarchical routing (ICCHR) [26], Multiple-Attribute Decision-Making (MADM) [27], Probabilistic Buckshot-Driven Cluster Head Identification (PB-RESHM) [23], Energy Aware Distance-based Cluster Head selection and Routing (EADCR) protocol [28], Spider Monkey Optimization (SMO), Energy-efficient Cluster Head Identification (SSMOECHS) [29], Group Search Ant Lion with Levy Flight (GAL-LF) [30], Grasshopper Optimization Algorithm (GOA) [31], Probabilistic Cluster Head Selection (LEACH-PRO) [32], heterogeneous Modified Grey Wolf Optimizer (HMGWO) [33], Fibonacci Based Trimet Graph Optimization (FBTGO) [34], and fitness-value-based improved GWO (FIGWO) [35], and are used in identifying the CH and exchanging the data with the BS. Optimizing the selection of the CH in a wireless sensor network involves prioritizing the node with the highest residual energy while considering the minimum energy consumption required by the communication mechanism. This will facilitate the transmission of a greater amount of data simultaneously by the CH. Energy or distance are often employed as the primary identifiers of the CH. Individual nodes with significant leftover resources and energy with low operating costs are the best candidates for cluster heads. Each round assigns an updated index value to the appropriate cluster head, relying on the leftover resources. Existing indices are updated to reflect the current best CH globally in the network.

### 2.1. Implementation Environment

The proposed model for precision agriculture is implemented in the simulation environment using the CupCarbon simulator, a discrete-event-driven model installed in a Windows environment. The Cupcarbon simulator is publicly available [36]. The network events are periodically captured from the simulator. Table 2 summarizes the implementation environment in detail.

**Figure 1:**
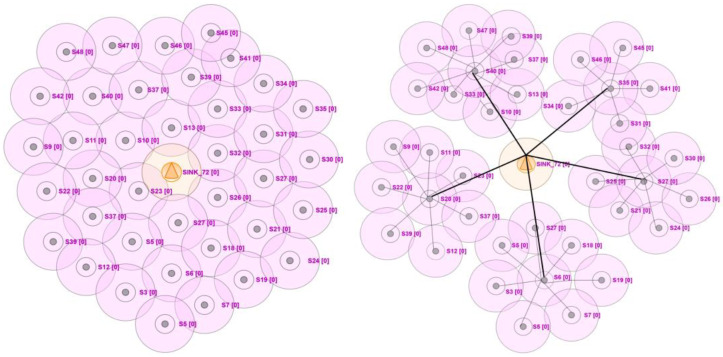
In sample screens of the implementation environment, the sink denotes the base station, and the circle around the sensors denotes the coverage area. Each node in the network is assigned a node number to differentiate.

This scenario takes place over a $100\times 100\;m^{2}$ elevation grid. A maximum transmission distance of 30 m may be detected in this case. The scenario is used to evaluate how well the given strategy works. The model was constituted over the same cohort of nodes executed for 2000 repetitions. The details of the simulated environment are discussed in Table 3. The sample screens of the simulation environment are presented in Figure 1.

### 2.2. Evaluation Metrics

The performance of the proposed energy-aware IoT architecture in PA is being assessed using various standard evaluation metrics like Energy consumption, network latency, mean network lifetime, bandwidth consumption, and the node’s communication fitness. The significance of each evaluation metric mentioned above is discussed along with the formula.

#### 2.2.1. Energy Consumption

Energy consumption is critical in determining the sensor network’s longevity. Each node in the network has leftover energy connected with it. The energy is utilized for communicating data among other nodes across the network. The longest-lasting nodes may designate the nodes with the most energy resources as the local head for data exchange with the BS. Energy consumption is a statistic used to assess network performance. Topological adjustments, such as the location of the linked BS and the sum of links associated with the node that will minimize the energy demand enhance the network’s sustainability. The energy usage for data exchange with BS is calculated using the following Equation (1) for direct communication. Nodes that rely on the other nodes would rely on the other nodes to exchange the data [37].

(1) $$energy_{dir_{t}}\left(dst\right)=d_{e}+d_{dc}dst^{2}$$

(2) $$energy_{exc_{t}}\left(dst\right)=d_{e}+\sum_{x=0}^{n}d_{e_{x}}dst^{4}$$

From the above equations,
$energy_{dir_{t}}\left(dst\right)$The energy needed to push the data directly to BS$energy_{exc_{t}}\left(dst\right)$The energy required for transferring the data through a neighboring node$d_{e}$Amount of power the intermediate relay nodes use during a data transfer.$d_{dc}$Amount of energy required for direct transfer of the data to BS.dstthe distances measured among the corresponding node and the destination nodenThe adjacent nodes that facilitate the transmission of data.

#### 2.2.2. Mean Network Lifetime

The mean network lifetime is another crucial statistic in assessing network design performance. It is always desired to have a network with a greater network lifetime. The sum of nodes in the network determines it. The network lifetime over the time stamps of the range 1 to n is assessed using Equation (3) [38].

(3) $$n_{l}=\sum_{tim=1}^{n}\frac{1}{\pi \left(2\;\times \;\left(\frac{d_{bs}}{n_{c}}\right)+1\right)\times n_{c}{}^{2}\times d_{n}}\times \frac{t_{n}-\pi \;\times \;d_{bs}{}^{2}\times d_{n}}{t_{n}}$$

where the notations used are as follows
$t_{n}$Denotes the total number of nodes in the network.$d_{n}$The nodes density within the network.$d_{bs}$The distance between the corresponding node to the BS.$n_{c}$Denote the network coverage.$n_{l}$Denote the network’s lifetime.

#### 2.2.3. Bandwidth Consumption

Data are continually transferred between network nodes and the BS, leading to data exchange bandwidth usage. The CH uses bandwidth while sharing data among sensor network nodes, which is desired to be minimum [39]. The formula for the bandwidth consumption is identified by

$$B_{c}$$

is shown in Equation (4).


(4) $$B_{c}=\;TA_{b}-\sum_{i=0}^{p}B_{r_{i}}\;$$

The variable $TA_{b}$ in the above equation denotes the total allotted bandwidth and the variable $B_{r}$ designates the bandwidth required at each instance i in the network. The bandwidth requirement is assessed based on the sum of spike packets passing through the node at an instance of time concerning bits per packet as shown in Equation (5).

(5) $$B_{r}=\;\frac{S_{p}\;\times \;b_{p}}{I_{t}}$$

where the notations used are as follows
$S_{p}$the sum of spike packets.$I_{t}$Instance of time.$b_{p}\;$Bits per packet.

#### 2.2.4. Node’s Communication Fitness

The communication fitness of a node is one of the indications used to determine if it can function as a CH. At some stage in the network, the node with appropriate communication fitness is identified as competent to become the CH. The fitness of node communication is tested regularly. The evaluated value aids in determining the best cluster head node for data transmission among nodes and the linked BS [40]. The formula for assessing the node’s fitness is shown in Equation (6).

(6) $$f_{n}=1-min\left(1,\sum_{a}\sum_{b}\left(max\left(0,\frac{x_{ab}-e_{t}}{e_{t}}\right)\right)/t_{n}\right)$$

where the notations used are as follows
$x_{ab}$ Represents the energy corresponding to the internal node communication.$e_{t}$ Energy threshold at the instance.$t_{n}$ Total number of nodes in the network. 

#### 2.2.5. Network Latency

Network latency refers to the time it takes to exchange data among nodes and the BS, i.e., connected BS, through CH. Network latency is intended to be as low as possible for speedier data interchange. If the packet must be transferred between numerous cluster heads before reaching the BS, the delay will grow as the number of data packets is exchanged. Each CH must wait until all the packets are processed, which includes propagation and queuing delays. In general, network latency is measured in terms of time delay associated with the distance the packet travels at a determined transmission rate to the size of the packet at the specified transmission rate [41]. The formula for network latency over the transmission rate $T_{r}$ is determined based on Equation (7). The propagation delay associated with the network is shown in Equation (8), and the Serialization delay is shown in Equation (9).

(7) $$n_{l}=\frac{p_{d}}{S_{d}}$$

(8) $$p_{d}=\;\frac{dst}{T_{r}}$$

(9) $$S_{d}=\frac{b_{p}}{T_{r}}$$

### 2.3. Architecture Assumptions

Several network assumptions are emphasized before discussing the technical aspects of the recommended Energy-aware IoT Framework for precision agriculture. The hypothesis is listed in bullet points as follows.

- There are divergent sensor nodes scattered in each network region for real-time monitoring.
- The regions in the network are circular size areas relying on the network coverage of the nodes.
- The nodes and the BS are fixed upon deploying them to establish the network.
- All the communication links among the sensors and sensor nodes to the BS are symmetric and bi-directional.
- Each network node differs concerning the availability of the residual energies like batter life, processing capabilities, and available memory.
- Based on the leftover energy reserves, nodes are classified as low, sustainable, or high.
- Each region in the network would have multiple clusters, and each would have multiple sensor nodes. Among those sensor nodes in the cluster, there would be one CH that is responsible for the exchange of the data.

## 3. Energy Aware Software Defined Network model

**Figure 2:**
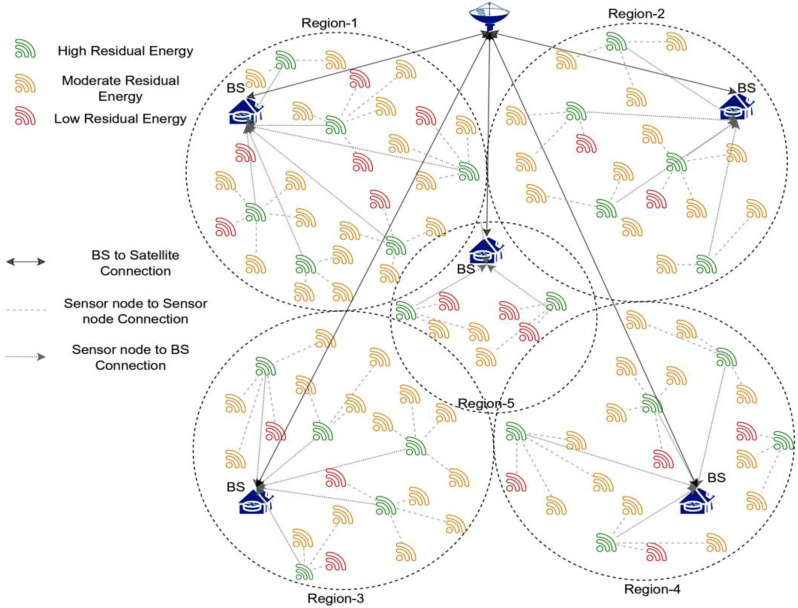
Image representing the connections among various devices in the network.

In each search space, the suggested model is non-dominant. Depending on available resources, nearby nodes, proximity to the corresponding BS, and associated network maintenance expenses, CHs are chosen. The proposed approach for sensor networks consists of two phases. At first, we consider energy, distance, and latency while selecting a cluster’s heads. In the next phase, the index at each cluster head is updated in line with the local and global best residual energies, and the indexes and the nodes are updated. The present subsection of the suggested framework provides an in-depth description of the framework. The connection among the sensor node, CHs, and BS is shown in Figure 2.

### 3.1. Initial Selection of the Cluster Head

The initial selection of the CH is based on multiple factors considered in choosing the optimal CH in the cluster. Choose the node with the most available residual and lowest energy consumption to find the most suitable CH. It will enable the CH to transmit more data packets. Energy and distance are the main determinants in locating the CH. It is desired to consider the CH close to the BS, as it would consume less energy to push the data to the BS, and the communication latency can also be minimized [42]. The CH-index is used in determining the significance of the CH. The node that has the highest CH-Index would be assumed to be the optimal node for being a cluster head. The energy metric for overall communication is identified by $E_{c}$ for p  packets over a distance d, from the node to the BS is shown in Equation (10) [43].

(10) $$E_{c}=E_{int}-\left\{\left(I_{e}\times p\right)+\left(E_{t}\times p\times d^{2}\right)\right\}\;$$

where the notations used are as follows
$I_{e}$Implied energy$E_{t}$The energy needed to push the data from the node to BS

The formula for implied energy is shown in Equation (11) [44].

(11) $$I_{e}=\;E_{td}+E_{ta}$$

where the notations used are as follows
$E_{td}$ The total energy deployed to exchange the p packets among the sensor node and the BS$E_{ta}$ The energy needed for data time aggregation

The total energy is measured as recognized by the notation $E_{tot}$, which also includes the amount of idle time recognized by $E_{i}$ of the node as shown in Equation (12).

(12) $$E_{tot}=E_{i}+E_{c}$$

The distance measure is assessed between the CH and the BS using the Euclidean distance measure. The Euclidean distance measure is generally used in spatial image processing techniques [45]. The same distance measure is used in measuring the distance among the coordinates in terms of measuring metrics, i.e., meters [46]. The notation $d_{m}$ recognize the corresponding formula for the distance measure as shown in Equation (13).

(13) $$d_{m}=\sqrt{{\left(m_{y}-n_{y}\right)}^{2}+{\left(m_{x}-n_{x}\right)}^{2}}$$

In the above equation, the $\left(m_{x},m_{y}\right)$ are the coordinates associated with the CH, and the coordinates $\left(n_{x},n_{y}\right)$ correspond to the BS. The delay among the nodes is the other significant parameter that is considered. The latency is proportional to the density of nodes within the cluster. As a result, the count of cluster nodes should be reduced to minimize the delay. The corresponding formula is shown in Equation (14).

(14) $$delay=\frac{max_{i=1}^{C_{n}}\left(CH_{i}\right)}{c_{n}}$$

In the above equation, the numerator $max_{i=1}^{C_{n}}\left(CH_{i}\right)$ denotes the maximum delay associated with the CH and the notation $c_{n}$ designates the sum of nodes in the cluster. The CH-index is measured based on all the above measures and the residual energy availability. The formula for the CH-index is shown in Equation (15).

(15) $$CH_{index}=\frac{R_{e_{i}}}{\frac{1}{c_{n}}\sum_{j=1}^{c_{n}}R_{e_{j}}}+\left(\omega_{x}\times E_{c}\right)+\left(\omega_{y}\times d_{m}\right)+\left(\omega_{z}\times delay\right)$$

**Figure 3:**
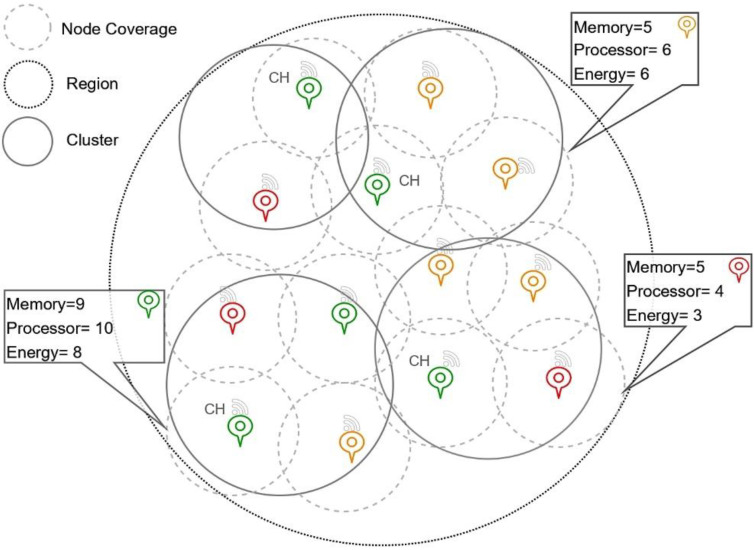
Image representing the node deployment and network topology.

From the above equation, the notations $\omega_{x},\omega_{y},\omega_{z}$ designated the weights associated with each of the features. The weight $\omega_{x}$ is associated with the communication energy metric, and the weight $\omega_{y}$ corresponds to the distance measure; the associated values are assumed to be in the range of 0 and 1. The weight associated with the delay, i.e., $\omega_{z}$ is considered 0.2 in the earlier studies. The deployment of the sensors and the network topology can be seen in Figure 3.

### 3.2. Updating of the CH-Index

The next phase of the proposed model is to update the CH-index values, which would assist in identifying the optimal CH in the current iteration. The index of the CH is being updated using the spider monkey optimization model. The updated CH-index value based on the SMO algorithm is used in the successive rounds of the network lifetime. The SMO approach considers the cluster heads’ local and global best CH-index values. During the initiation phase, the initial fitness of the search space is determined based on resided energy index, delay, and distance measures. In the successive phases, the CH-index values are considered in optimizing the index values. The corresponding objective function for updating CH-index is shown in Equation (16).

(16) $$CH_{index}=CH_{index}+\left(I_{lb}-CH_{index}\right)\times rand\left(0,1\right)+\left(CH_{pb}-CH_{index}\right)\times rand\left(-1,1\right)$$

In the above equation, the notation $I_{lb}$ represents the index value of the local best node in the cluster, especially when a node other than the CH may have a better index value based on the current environmental conditions. The function $rand\left(0,1\right)$ is to confine the range of values between 0 and 1 and similarly for the function $rand\left(-1,1\right)$. The notation $CH_{pb}$ designates the node’s perturbation rate based on underlying factors. The value of the CH-Index would be updated based on the index value of the global best. The global best is recognized as the node with the highest CH-Index value. The corresponding function to update the index values based on the global best is shown in Equation (17).

(17) $$CH_{index}=CH_{index}+\left(CH_{gb}-CH_{index}\right)\times rand\left(0,1\right)+\left(I_{lb}-CH_{index}\right)\times rand\left(-1,1\right)$$

In the above equation, the notation $CH_{gb}$ designates the global best value of the index values. The assessed CH-index is used in updating the CH.

The fitness of the cluster is assessed every time to decide if the cluster could be retained for the next successive rounds or to merge the nodes in the cluster with the neighboring clusters. The fitness of the cluster is identified by $f_{c}$ and is assessed using the formula as shown in Equation (18).

(18) $$f_{c}=\frac{1}{\sum_{i}^{n_{c}}\sum_{j}^{n_{i}}\sqrt{\left|CH_{index}-M_{index}\right|}}$$

From the above equation, the notation $n_{c}$ denotes the sum of clusters in the network, and the notation $n_{i}$ denotes the sum of corresponding nodes within the cluster. The mean index values of all the nodes in the cluster are identified by $M_{index}$. Based on the value of the cluster fitness, the clusters in the network are retained for subsequent rounds of the transmission network. The Algorithm 1 for the CH selection is shown below.


**Algorithm 1: CH selection**

*Input:*


   Number Nodes: 100

   Initial Energy: 100 mW

   Number of rounds: 2000


*Output:*


   Assessment: Network Lifetime, Network Delay, Network Throughput   **Start**

*Function:*
**Initial_CH-Index()**

 **for** 1 to n do // n denotes max nodes in the network


$\;\mathrm{Calculate}\;E_{c}$

$//E_{c}$ denotes energy consumption


$\;\mathrm{Calculate}\;d_{m}$

$//d_{m}$ denotes the distance measure


   Calculate delay

//communication delay


$\;\mathrm{Approximate}\;(\omega_{x},\omega_{y},\omega_{z})$


$\;\mathrm{Initial}\_\mathrm{CH}-\mathrm{index}(E_{c}$, $d_{m}$*,*delay*,*$\omega_{x}$*,*$\omega_{y}$*,*$\omega_{z}$)
       **return** (CH-index value)
 **end for**
**while** (round < 2000) do
   *Function:*
**Update CH-Index()**
     **for** 1 to n
**do**


$\;\mathrm{Identify}\;I_{lb}$




$\;\mathrm{Identify}\;CH_{gb}$




$\;\mathrm{Calculate}\;CH_{pb}$


$\;\mathrm{Update}\_\mathrm{CH}-\mathrm{Index}(I_{lb}$,$CH_{gb}$, $CH_{pb}$)
          **return** (CH-index value)
   **end for**

*Function: Cluster_fitness()*



$\;\mathrm{for}\;1\;\mathrm{to}\;n_{c}$

**do**
$//n_{c}$ denotes the number of clusters


$\;\mathrm{for}\;1\;\mathrm{to}\;n_{i}$

**do**
$//n_{i}$ denotes the number of nodes in the cluster


$\;\mathrm{Calculate}\;M_{index}$

$//M_{index}$ mean of all node index
$\;\mathrm{Cluster}\_\mathrm{fitness}(M_{\mathrm{index}}$, ${\mathrm{CH}}_{\mathrm{index}}$)
       **return** (Cluster_fitness value)
  **end for**
 **end** for
**Update** Clusters    **Return** (Lifetime, Delay, Throughput)  **Stop**

## 4. Results and Discussion

**Figure 4:**
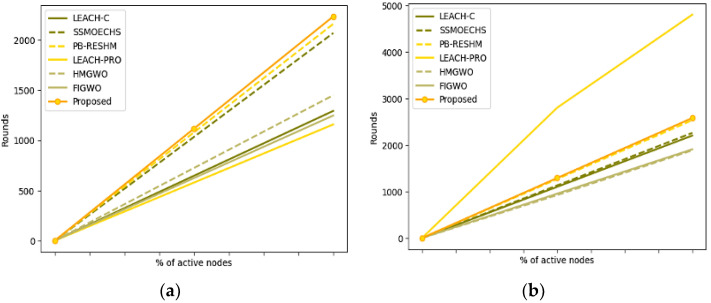
(**a**) Graphs representing the analysis of the First Node Die (**b**) Graphs representing the Last Node Die.

Regarding network availability, throughput, remaining energy, energy usage, and mean network lifetime, the suggested software-driven network model is carefully studied compared to the findings of various advanced network models. The first connection creation and node list update take 845-time units. The proposed software-driven network model is compared to current models using a comparable experimental setting. In the current study, various models like LEACH, PB-RESM, SSMOECHS, Two-Tier Clustering-based Data Aggregation (TTCDA) [47], and Energy-efficient Clustering Data Aggregation (EECDA) [48] are considered in the evaluation of the proposed model. The network life is the metric measured in terms of time units, which is generally analyzed against the percentage of the active nodes in the network along with measures like First Node Die (FND) and Last Node Die (LND). The FND is the metric that denotes when the first node turned inactive in the network, and the LND denotes the last node that turned inactive in the network. Either of the metrics would assist in analyzing the network lifetime. The network lifetime performance is shown in Table 4 on executing for 3000 rounds. The corresponding graphs generated from the experimental values of network lifetime are shown in Figure 4.

**Figure 5:**
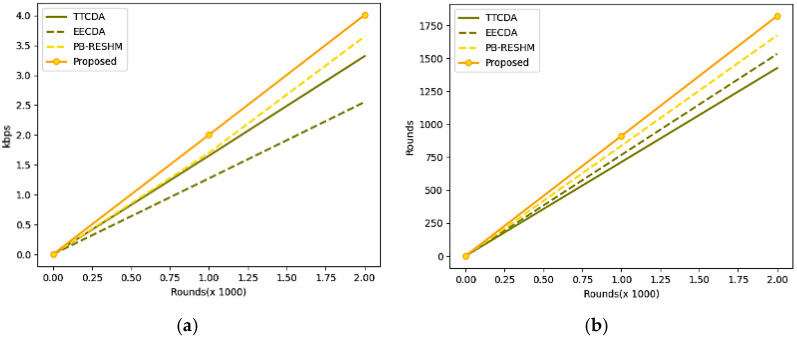
(**a**) Graphs representing the analysis of network throughput, (**b**) Graph representing the analysis of network lifetime.

The model’s performance is assessed concerning throughput and network lifetime metrics. It is desired that the network throughput is always desired to be high, which determines the sum of packets that are successfully delivered. The study’s throughput is measured as kilobits per second (kbps). The network lifetime is measured in time units, which elucidates the availability of the nodes in the network, which is also desired to be high. The lifetime metrics are evaluated as the total rounds of the network to deliver the packet without significant loss successfully. The experimental values of network throughput and lifetime are shown in Table 5. The corresponding graphs of network throughput and network lifetime are shown in Figure 5.

**Figure 6:**
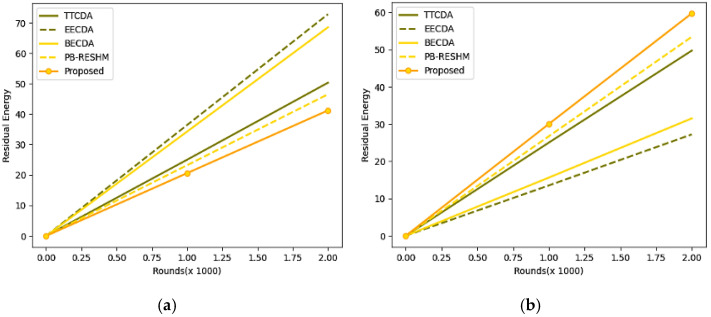
(**a**) Graphs representing the analysis of energy utilization for 2000 rounds, (**b**) Graphs representing the analysis of leftover residual energy.

The other metrics, like energy consumption and leftover energy at the stable homogeneous network, are discussed in Table 6. From the metrics mentioned above, leftover energy is the metric that determines the remaining residual energy in the network upon successful transmission of the data, which was desired to be high. Energy utilization is the metric that presents the overall energy consumption for network maintenance and data exchange, which is desired to be a minimum. The energy parameter in the current study is measured in terms of milliwatts (mW). The corresponding energy utilization and leftover energy-related graphs are shown in Figure 6.

**Figure 7:**
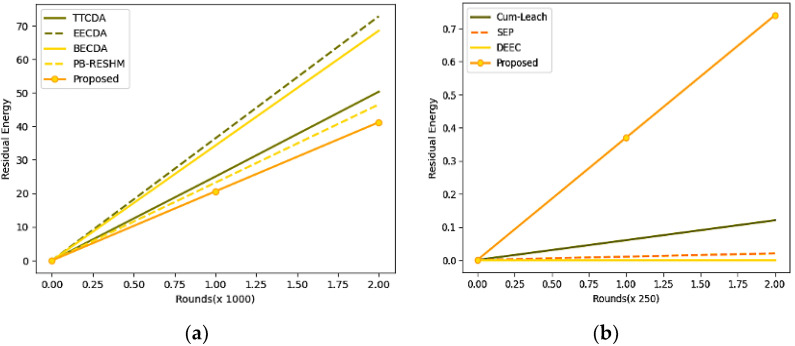
(**a**) Graphs representing the analysis of energy utilization, (**b**) Graph representing the analysis of leftover residual energy.

For better analysis of the efficiency, the model is analyzed with other cutting-edge techniques like cumulative low-energy adaptive clustering hierarchy-LEACH(Cum_LEACH) [49], stable election protocol (SEP) [50], and distributed energy-efficient clustering (DEEC) [51]. The model is evaluated with an initial energy of 50 mW instead of 100 mW as the initial simulated energy for the other evaluations. Table 7 shows the model’s performance, and leftover energy and consumption are reported in milliwatts to preserve consistency throughout the study. The experimental values are evaluated for 500 rounds. The energy consumption and leftover energy graphs are shown in Figure 7, with reduced initial energy over 500 rounds.

**Figure 8:**
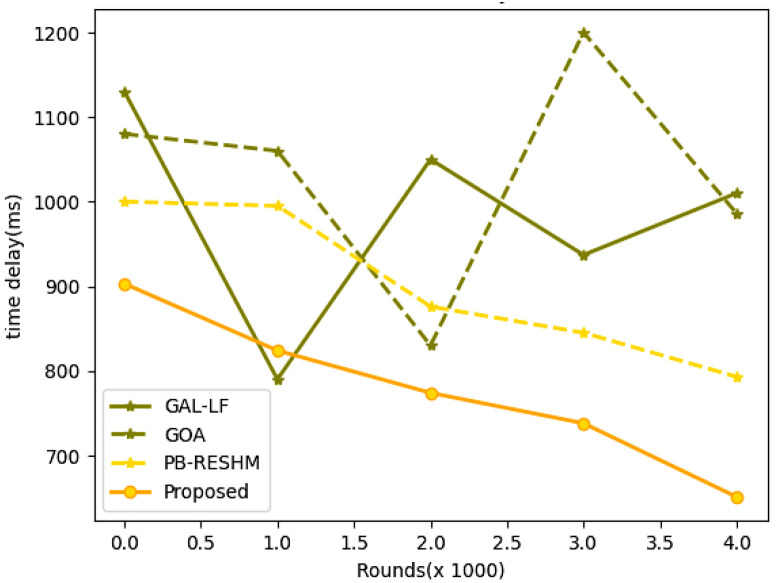
Graphs representing the network delay over multiple rounds.

The experimental results in Table 4 and Table 5 demonstrate that the proposed model exhibits superior performance compared to the advanced models employed for comparison. The proposed model has resulted in better network throughput, lifetime, and leftover residual energy. The energy utilization is retained to be minimal compared to the other models that are considered in the evaluation process. The other significant evaluation metric is the computational delay in the network, which is assessed for each round. The analysis is made over multiple rounds. The experimental results on delay evaluation are presented in Table 8, and the corresponding graph on network delay is shown in Figure 8.

The proposed model has a minimal time delay at all rounds from starting till the end of the network’s analysis. The network with minimal would have a faster response to the service request, and the network would be more productive. The proposed network procedure has been shown to perform well across several assessment parameters. The proposed model has approximately 26% times better node sustainability in the network than the other existing models. It can be observed from the results obtained that the time delay at the 2000th round of the proposed model is approximately 18% lesser than the PB-RESHM, which is a model that holds the least time delay among the existing contemporary models. The energy utilization of the model is approximately 14% better than the other state-of-art models, and the model holds approximately 10% more residual energy than the other existing models. The comparison in the study is made across multiple approaches, and the experimental values of the state-of-art models are acquired from the previous publications to ensure the values are authentic, resulting in the change of approaches over multiple parameters.

## 5. Conclusions

The studies have determined that the WSNs are a crucial component of remote sensing for smart agricultural systems, allowing for better monitoring, temperature monitoring, irrigation system monitoring, and water supply monitoring. It’s crucial because it facilitates interaction between various network entities in the intelligent agricultural ecosystem. A WSN is made up of nodes that are in constant contact with one another and with a BS. The sensors’ topology management, mapping, and storage, as well as their battery life, all have their drawbacks. Due to these barriers, the efficiency of the intelligent farm system has been diminished. The proposed energy-aware model is robust in establishing and maintaining the network with reasonable lifetime, delay, and energy consumption. The experimental analysis with various contemporary models has proven that the proposed approach has outperformed network management. The unique way of choosing the initial CH based on the underlying node capabilities, updating the cluster head index based on the local and global best indexed, and assessing the cluster fitness to retain the cluster in subsequent rounds would assist in better network establishment and maintenance.

The forthcoming strategy involves utilizing and establishing auto-encoder technology to deploy sensors and prioritize feature significance in the cluster head selection process within the network. The futuristic evaluations may consider evaluating against the network with a broader area with dense nodes for precise analysis of the network’s robustness. The network performance can be further assessed by incorporating the temporary support nodes to improve the network’s lifetime.

## Figures and Tables

**Table 1:** Summary of various state-of-art models in WSN for real-time data exchange.

Approach	Highlights	Limitations
Sentek commercial sensor [9]	Affordable precision agriculture employing a low-cost soil moisture sensor that works on Lora WAN	The model is efficient in low-data rate communication. This technology is feasible to implement over limited sensors. However, multiple other meteorological sensors would impact the performance of the network model.
WiLD network [11]	Wi-Fi long-distance network is designed to implement over a fog technology that is energy efficient, has low latency, and has substantial throughput	The model is efficient for fog technology, but the technology needs additional routers, hubs, and gateways for data storage and processing system.
SMAIoT network [16]	Smart agricultural IoT is efficient in acquiring and analyzing real-time data from various in-expensive sensors automating tasks, such as irrigation, fertilizers, insect identification, and pesticide spraying.	The study has limitedly analyzed the latency and the network’s sustainability, which are crucial in applications like PA.
LoRaWAN network [17]	Long-Range Wide-Area Network is efficient in applications like healthcare and smart agriculture domains. The data among the sensor’s devices are a well-secured manner.	The technology is feasible for low data rate applications and applications where network latency is not a crucial parameter.
LEACH [22]	Low-energy adaptive cluster hierarchy models are one of the energy-efficient models used in WSN technology for interconnecting multiple nodes in the network.	The model does not take the distance measures like node-node and node-BS distances are not considered while assessing the energy models, and residual energy is not considered when selecting the CH.
PB-RESHM [23]	Probabilistic Buckshot-Driven Cluster Head Identification is an efficient model for choosing the optimal cluster head to exchange data in the WSN.	The proposed model selects the optimal CH provided the CH in the initial round is efficiently chosen, as the CH in subsequent rounds relies on the CH in the current round.

**Table 2:** Details of the Implementation Environment.

Environment Details	Specifications
Machine	HP Pavilion series
Processor	Intel Core-i5 12th Generation
Operating System	Windows 10
Architecture	X64-bit
Memory	16 GB DDR4-3200 MHz RAM
Software	CupCarbon Simulator
Simulator version	V5.2
Supporting software	Java JDK 1.8

**Table 3:** Details of the simulation Environment.

Environment Details	Specifications
Topological Area (In meters)	$100\times 100\;m^{2}$
Location of BS	50×50
Number of Nodes	100
Initial Energy of Node	100 mW
Number of Base Stations	1
Stationary or mobile	Stationary
Radio electronic energy	50 nJ/bit
Message length [node to CH]	2800-bits
Message length [CH to BS]	6400-bits
Length of control packet	200-bits
Number of rounds of packet transmission	500/2000/3000
Limit of transmission distance	50 m
Center frequency (GSM900)	950 MHz
Intra-cluster routing	Single Hop

**Table 4:** Experimental values of network lifetime.

Approaches	FND	% of Nodes Alive in the Network				LND
		90	70	50	30	
LEACH-C	1294	1515	1695	1843	1976	2201
SSMOECHS	2071	2172	2218	2242	2250	2259
PB-RESHM	2162	2236	2309	2385	2439	2532
LEACH-PRO	1159	NA	NA	1720	NA	4800
HMGWO	1450	NA	NA	1675	NA	1884
FIGWO	1248	NA	NA	1612	NA	1906
Proposed Approach	2237	2268	2330	2397	2470	2579

**Table 5:** Experimental values of Network Throughput and lifetime.

	Network Throughput (kbps)	Network Lifetime
TTCDA	3.32	1425
EECDA	2.55	1532
PB-RESHM	3.65	1673
**Proposed model**	4.01	1820

**Table 6:** Experimental values of Energy utilization and Leftover Residual Energy.

Approaches	Energy Utilization (mW)	Leftover Residual Energy (mW)
TTCDA	50.3	49.7
BECDA	68.5	31.5
EECDA	72.8	27.2
PB-RESHM	46.5	53.4
Proposed model	41.2	59.7

**Table 7:** Experimental values of Energy utilization and Leftover Residual Energy with reduced initial energy over 500 rounds.

Approaches	Energy Utilization (mW)	Leftover Residual Energy (mW)
Cum_LEACH	13.76	0.12
SEP	13.78	0.02
DEEC	13.65	0
Proposed model	11.73	0.74

**Table 8:** Experimental values of time delay in milliseconds at various rounds of the simulation.

Approaches	Rounds				
	1	500	1000	1500	2000
GAL-LF	1130	790	1050	937	1010
GOA	1080	1060	830	1200	986
PB-RESHM	1000	995	876	843	793
Proposed Approach	903	824	774	738	651

## Data Availability

Not applicable.

## References

[1] Zhang R., Li X. (2021). Edge Computing Driven Data Sensing Strategy in the Entire Crop Lifecycle for Smart Agriculture. Sensors.

[2] Liu J., Xiang J., Jin Y., Liu R., Yan J., Wang L. (2021). Boost Precision Agriculture with Unmanned Aerial Vehicle Remote Sensing and Edge Intelligence: A Survey. Remote Sens..

[3] Akhtar M.N., Shaikh A.J., Khan A., Awais H., Bakar E.A., Othman A.R. (2021). Smart Sensing with Edge Computing in Precision Agriculture for Soil Assessment and Heavy Metal Monitoring: A Review. Agriculture.

[4] Prachi S., Prem C.P., George P.P., Andrew P., Prashant K.S., Nikos K., Khidir A.K.D., Yangson B. (2020). Hyperspectral remote sensing in precision agriculture: Present status, challenges, and future trends. Earth Observation, Hyperspectral Remote Sensing.

[5] Lampridi M.G., Kateris D., Vasileiadis G., Marinoudi V., Pearson S., Sørensen C.G., Balafoutis A., Bochtis D. (2019). A Case-Based Economic Assessment of Robotics Employment in Precision Arable Farming. Agronomy.

[6] Liakos K.G., Busato P., Moshou D., Pearson S., Bochtis D. (2018). Machine learning in agriculture: A review. Sensors.

[7] Mottaleb K.A. (2018). Technology in Society Perception and adoption of a new agricultural technology: Evidence from a developing country. Technol. Soc..

[8] Haseeb K., Ud Din I., Almogren A., Islam N. (2020). An Energy Efficient and Secure IoT-Based WSN Framework: An Application to Smart Agriculture. Sensors.

[9] Placidi P., Morbidelli R., Fortunati D., Papini N., Gobbi F., Scorzoni A. (2021). Monitoring soil and ambient parameters in the iot precision agriculture scenario: An original modeling approach dedicated to low-cost soil water content sensors. Sensors.

[10] Hsu T.-C., Yang H., Chung Y.-C., Hsu C.-H. (2020). A Creative IoT agriculture platform for cloud fog computing. Sustain. Comput. Inform. Syst..

[11] Ahmed N., De D., Hussain M.I. (2018). Internet of Things (IoT) for Smart Precision Agriculture and Farming in Rural Areas. IEEE Internet Things J..

[12] Trilles S., Torres-Sospedra J., Belmonte Ó., Zarazaga-Soria F.J., González-Pérez A., Huerta J. (2020). Development of an open sensorized platform in a smart agriculture context: A vineyard support system for monitoring mildew disease. Sustain. Comput. Inform. Syst..

[13] Kwok J., Sun Y. (2018). A smart IoT-based irrigation system with automated plant recognition using deep learning. Proceedings of the 10th International Conference on Computer Modeling and Simulation—ICCMS2018.

[14] Ratnaparkhi S., Khan S., Arya C., Khapre S., Singh P., Diwakar M., Shankar A. (2020). Smart agriculture sensors in IOT: A review. Mater. Today Proc..

[15] Jawad H.M., Nordin R., Gharghan S.K., Jawad A.M., Ismail M. (2017). Energy-Efficient Wireless Sensor Networks for Precision Agriculture: A Review. Sensors.

[16] Jani K.A., Chaubey N.K. (2021). A Novel Model for Optimizing Resource Utilization in Smart Agriculture System Using IoT (SMAIoT). IEEE Internet Things J..

[17] Almuhaya M.A.M., Jabbar W.A., Sulaiman N., Abdulmalek S. (2022). A Survey on LoRaWAN Technology: Recent Trends, Opportunities, Simulation Tools and Future Directions. Electronics.

[18] Basford P.J., Bulot F.M.J., Apetroaie-Cristea M., Cox S.J., Ossont S.J. (2020). LoRaWAN for Smart City IoT Deployments: A Long Term Evaluation. Sensors.

[19] Rahman H.U., Ahmad M., Ahmad H., Habib M.A. LoRaWAN: State of the Art, Challenges, Protocols and Research Issues. Proceedings of the 2020 IEEE 23th International Multitopic Conference (INMIC).

[20] Pinto-Erazo A.M., Suárez-Zambrano L.E., Mediavilla-Valverde M.M., Andrade-Guevara R.E., Rocha Á., López-López P.C., Salgado-Guerrero J.P. (2022). Introductory Analysis of LoRa/LoRaWAN Technology in Ecuador. Communication, Smart Technologies and Innovation for Society. Smart Innovation, Systems and Technologies.

[21] Vij A., Vijendra S., Jain A., Bajaj S., Bassi A., Sharma A. (2020). IoT, and machine learning approaches for automation of farm irrigation system. Proc. Comput. Sci..

[22] Mahapatra R.P., Yadav R.K. (2015). Descendant of LEACH Based Routing Protocols in Wireless Sensor Networks. Procedia Comput. Sci..

[23] Srinivasu P.N., Panigrahi R., Singh A., Bhoi A.K. (2022). Probabilistic Buckshot-Driven Cluster Head Identification and Accumulative Data Encryption in WSN. J. Circuits Syst. Comput..

[24] Shakeel A., Srinivasu P.N., Gupta M. (2023). Future perspectives of AI-driven Internet of Things. Aiot Technologies and Applications for Smart Environments.

[25] Han B., Ran F., Li J., Yan L., Shen H., Li A. (2022). A Novel Adaptive Cluster Based Routing Protocol for Energy-Harvesting Wireless Sensor Networks. Sensors.

[26] Wu H., Zhu H., Zhang L., Song Y. (2019). Energy Efficient Chain Based Routing Protocol for Orchard Wireless Sensor Network. J. Electr. Eng. Technol..

[27] Lekhra J., Kumar A., Kumar A. (2022). An approach based on modified multiple attribute decision making for optimal node deployment in wireless sensor networks. Int. J. Inf. Technol..

[28] Panchal A., Singh R. (2022). EADCR: Energy Aware Distance Based Cluster Head Selection and Routing Protocol for Wireless Sensor Networks. J. Circuits Syst. Comput..

[29] Lee J.-G., Chim S., Park H.-H. (2019). Energy-Efficient Cluster-Head Selection for Wireless Sensor Networks Using Sampling-Based Spider Monkey Optimization. Sensors.

[30] Dattatraya K.N., Rao K.R. (2020). Maximising network lifetime and energy efficiency of wireless sensor network using group search Ant Lion with Levy Flight. IET Commun..

[31] Saremi S., Mirjalili S., Lewis A. (2017). Grasshopper optimisation algorithm: Theory and application. Adv. Eng. Softw..

[32] Yousif Z., Hussain I., Djahel S., Hadjadj-Aoul Y. (2021). A Novel Energy-Efficient Clustering Algorithm for More Sustainable Wireless Sensor Networks Enabled Smart Cities Applications. J. Sens. Actuator Netw..

[33] Zhao X., Ren S., Quan H., Gao Q. (2020). Routing Protocol for Heterogeneous Wireless Sensor Networks Based on a Modified Grey Wolf Optimizer. Sensors.

[34] Amiripalli S.S., Bobba V., Srinivasu N.P. (2022). Design and Analysis of Fibonacci Based TGO Compared with Real-time Mesh using Graph Invariant Technique. Int. J. Sens. Wirel. Commun. Control.

[35] Zhao X., Zhu H., Aleksic S., Gao Q. (2018). Energy-Efficient Routing Protocol for Wireless Sensor Networks Based on Improved Grey Wolf Optimizer. KSII Trans. Internet Inf. Syst..

[36] CupCarbon IoT Simulator. https://cupcarbon.com/.

[37] Liu X., Wu J. (2019). A Method for Energy Balance and Data Transmission Optimal Routing in Wireless Sensor Networks. Sensors.

[38] Jerew O., Blackmore K., Liang W. (2012). Mobile Base Station and Clustering to Maximize Network Lifetime in Wireless Sensor Networks. J. Electr. Comput. Eng..

[39] Mantri D.S., Prasad N.R., Prasad R. (2015). Bandwidth efficient cluster-based data aggregation for Wireless Sensor Network. Comput. Electr. Eng..

[40] Mukase S., Xia K., Umar A. (2021). Optimal Base Station Location for Network Lifetime Maximization in Wireless Sensor Network. Electronics.

[41] Navin Dhinnesh A.D.C., Sabapathi T. (2022). Probabilistic neural network based efficient bandwidth allocation in wireless sensor networks. J. Ambient. Intell. Humaniz. Comput..

[42] Wu M., Li Z., Chen J., Min Q., Lu T. (2022). A Dual Cluster-Head Energy-Efficient Routing Algorithm Based on Canopy Optimization and K-Means for WSN. Sensors.

[43] Gurumoorthy S., Subhash P., Pérez de Prado R., Wozniak M. (2022). Optimal Cluster Head Selection in WSN with Convolutional Neural Network-Based Energy Level Prediction. Sensors.

[44] Son Y., Kang M., Kim Y., Yoon I., Noh D.K. (2020). Energy-Efficient Cluster Management Using a Mobile Charger for Solar-Powered Wireless Sensor Networks. Sensors.

[45] Srinivasu N.P., Rao S.T., Srinivas G., Reddy P.P.V.G.D. (2020). A Computationally Efficient Skull Scraping Approach for Brain MR Image. Recent Adv. Comput. Sci. Commun..

[46] Azad P., Sharma V. (2013). Cluster Head Selection in Wireless Sensor Networks under Fuzzy Environment. Int. Sch. Res. Not..

[47] Mantri D., Prasad N.R., Prasad R., Ohmori S. Two tier cluster based data aggregation (TTCDA) in wireless sensor network. Proceedings of the 2012 IEEE International Conference on Advanced Networks and Telecommunciations Systems (ANTS).

[48] Chao S., Wang R.-C., Huang H.-P., Sun L.-J. (2010). Energy efficient clustering algorithm for data aggregation in wireless sensor networks. J. China Univ. Posts Telecommun..

[49] Repuri R.K., Darsy J.P. (2023). Energy-Efficient LoRa Routing for Smart Grids. Sensors.

[50] Shrivastav K., Kulat K.D. (2020). Scalable energy efficient hexagonal heterogeneous broad transmission distance protocol in WSN-IoT Networks. J. Electr. Eng. Technol..

[51] Qing L., Zhu Q., Wang M. (2006). Design of a distributed energy-efficient clustering algorithm for heterogeneous wireless sensor networks. Comput. Commun..

